# Synthesis and Characterization of Nano Boron Nitride Reinforced Magnesium Composites Produced by the Microwave Sintering Method

**DOI:** 10.3390/ma6051940

**Published:** 2013-05-10

**Authors:** Sankaranarayanan Seetharaman, Jayalakshmi Subramanian, Khin Sandar Tun, Abdelmagid S. Hamouda, Manoj Gupta

**Affiliations:** 1 Department of Mechanical Engineering, National University of Singapore, 9 Engineering Drive 1, Singapore 117576; E-Mails: seetharaman.s@nus.edu.sg (S.S.); mpejs@nus.edu.sg (J.S.); 2 Technology Development Centre, ITE College Central, H1-01, 2 Ang Mo Kio Drive, Singapore 567720; E-Mail: sandar_k_tun@ite.edu.sg; 3 Mechanical and Industrial Engineering Department, Qatar University, Doha P.O. Box No. 2713, Qatar; E-Mail: hamouda@qu.edu.qa

**Keywords:** metal matrix composites, microwave sintering, mechanical properties, microstructure, X-ray diffraction, scanning electron microscopy

## Abstract

In this study, magnesium composites with nano-size boron nitride (BN) particulates of varying contents were synthesized using the powder metallurgy (PM) technique incorporating microwave-assisted two-directional sintering followed by hot extrusion. The effect of nano-BN addition on the microstructural and the mechanical behavior of the developed Mg/BN composites were studied in comparison with pure Mg using the structure-property correlation. Microstructural characterization revealed uniform distribution of nano-BN particulates and marginal grain refinement. The coefficient of thermal expansion (CTE) value of the magnesium matrix was improved with the addition of nano-sized BN particulates. The results of XRD studies indicate basal texture weakening with an increase in nano-BN addition. The composites showed improved mechanical properties measured under micro-indentation, tension and compression loading. While the tensile yield strength improvement was marginal, a significant increase in compressive yield strength was observed. This resulted in the reduction of tension-compression yield asymmetry and can be attributed to the weakening of the strong basal texture.

## 1. Introduction

In recent years, the research and development of new magnesium materials is receiving increased attention, as they exhibits tremendous application potential in the aerospace and transportation sectors, owing to their weight saving capabilities. Besides being light, Mg also possesses excellent damping characteristics, machinability and good castability. However, its poor ductility at room temperature due to the low symmetry and the paucity of slip systems in the hexagonal closed packed (HCP) structure restrict its extensive application [1,2,3,4].

In Mg, the thermomechanical processes, such as extrusion, results in a strong texture development aligning the basal planes strongly into the extrusion direction, which are highly unfavorable for basal slip to occur. Hence, the extruded Mg-materials exhibit poor ductility and different yield strengths under tension and compression. The compression yield stress is normally about 1/2 or 3/4 of the tension yield stress along the extrusion direction. Also, the tensile ductility of extruded Mg-based materials is limited, due to the difficulty in basal slip activation if the testing is carried out parallel to the extrusion direction [4,5,6,7]. Literature study reveals numerous attempts being made to improve the deformation behavior of Mg at room temperature [8,9,10,11,12]. In these studies, the property improvements are achieved primarily by the activation of non-basal slip systems/twinning, brought forth by texture modification through the addition of nanoscale reinforcements [8,9,10,11,12]. In this regard, boron nitride (BN) is an interesting material owing to its unique combination of properties, such as low density, high melting point, high thermal conductivity and high electrical resistivity [13,14]. Literature studies reveal the positive influence of boron nitride particles on the mechanical properties improvement in aluminum matrix composites [13,14]. However, in magnesium matrix composites, while the oxide reinforcements, such as Al_2_O_3_, Y_2_O_3_ and ZrO_2_, and the carbide reinforcements, like SiC in nanoscale, are extensively used by various researchers, no attempt has been made so far to synthesize nano-BN reinforced magnesium composites.

Alongside the development of new materials, unconventional processing techniques utilizing electromagnetic sources also result in energy savings, and in this regard, the microwave heating of metals has been proven as an efficient material processing technique [15]. Unlike directional microwave sintering, where the direction of heating is from inside to outside of the powder compact, the two directional rapid microwave sintering uses the combined action of microwaves and microwave-coupled external heating and has the major advantage of sintering the powder compacts at a relatively shorter period of time [15].

In the current work, nano-sized boron nitride particulates were incorporated in pure Mg through the powder metallurgy technique, using microwave sintering of the powder compacts. The effect of varying volume fractions of nano-BN addition on the microstructural and mechanical properties of pure Mg is investigated. The structure-property relationship is used to understand the observed mechanical behavior of the developed Mg/BN nanocomposites.

## 2. Results and Discussion

### 2.1. Synthesis

Pure Mg and its composites containing nano-BN particulates were successfully synthesized using powder metallurgy technique coupled with the microwave-assisted rapid sintering technique. The visual observation of the sintered billets and extruded rods did not reveal any defects and the successful synthesis of Mg materials without any macrostructural defects confirms the suitability of the process and process variables used in the study. The results of the characterization studies performed on the materials clearly indicate the feasibility of using microwave sintering to develop Mg composites [8,11,12,16].

### 2.2. Density and Porosity

The results of density and porosity measurements conducted on the developed Mg/BN nanocomposites are shown in Table 1. An increase in the experimental density values was observed due to the addition of BN-nanoparticulates, which is due to the relatively higher density of BN (2.98 g/cc) and, hence, increases with increasing BN addition. With regard to porosity, the obtained experimental values are relatively low in comparison to similar works, and this confirms the successful synthesis of near-dense materials with minimal porosity (≤0.38%) through the powder metallurgy method assisted with microwave sintering followed by hot extrusion [8,11,12,16].

**Table 1:** Results of density and porosity measurements.

S. No.	Material	BN (vol.%)	Theoretical density	Experimental density	Porosity
1	Pure Mg	0.00	1.7400	1.7365 ± 0.0011	0.20 ± 0.005
2	Mg-0.5BN	0.29	1.7436	1.7391 ± 0.0034	0.26 ± 0.001
3	Mg-1.5BN	0.86	1.7506	1.7458 ± 0.0014	0.21 ± 0.001
4	Mg-2.5BN	1.44	1.7579	1.7512 ± 0.0019	0.38 ± 0.004

### 2.3. Microstructure

**Figure 1:**
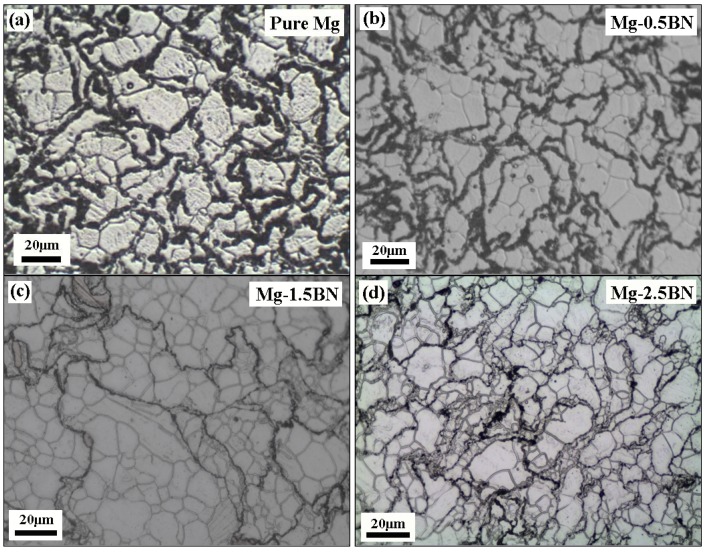
Optical micrographs showing the grain characteristics of: (**a**) pure Mg; (**b**) Mg-0.5BN; (**c**) Mg-1.5BN; and (**d**) Mg-2.5BN.

The grain characteristics and morphology of developed Mg materials are shown in Figure 1 and listed in Table 2. The microstructural characteristics (average grain size and aspect ratio) of pure Mg and Mg/BN composites based on grain size measurements conducted on the optical micrographs (Figure 1) are presented in Table 2. It revealed the unimodal distribution of grain size, which indicates one maxima for the grain size distribution, *i.e.*, maximum grains of the same size. This shows the complete recrystallization of Mg-matrix during extrusion [17]. Further, the addition of nanosized BN particulates resulted in marginal reduction of average matrix grain size from 28µ to 19µ (Table 2). However, considering the standard deviation, it was observed to be minimal and independent of the reinforcement volume fraction.

**Table 2:** Results of grain size measurements.

S. No.	Material	Grain size (µm)	Aspect ratio
1	Pure Mg	28.94 ± 5.86	1.61 ± 0.55
2	Mg-0.5BN	21.42 ± 2.67	1.54 ± 0.44
3	Mg-1.5BN	22.15 ± 1.92	1.67 ± 0.39
4	Mg-2.5BN	19.43 ± 3.43	1.56 ± 0.42

**Figure 2:**
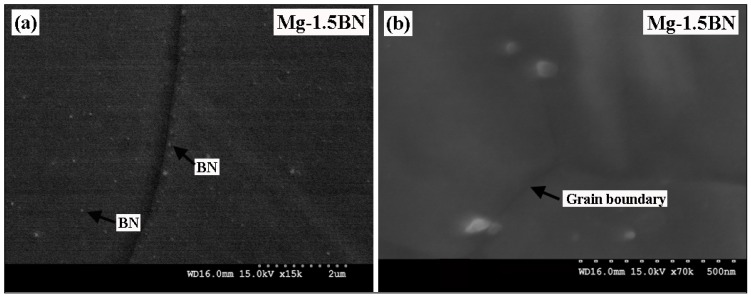
Representative SEM micrographs showing (**a**) the distribution of nano-boron nitride (BN) particulates; and (**b**) the interface between Mg-matrix and the nano-BN particulates in Mg-1.5BN composite.

The results of microstructural characterization studies conducted on the different Mg/BN composite formulations indicate a fairly uniform distribution of nano-BN particulates inside the Mg matrix, as well as near to the grain boundaries, as seen in Figure 2a. The observed uniform distribution of nano-BN particulates in the Mg matrix, as seen Figure 2, could be attributed to the following reasons: (i) closer density values between the Mg matrix (1.7 g/cc) and BN reinforcements (2.98 g/cc), resulting in lesser gravity assisted segregation problems during the blending, compaction and sintering process; (ii) suitable blending parameters used; and (iii) the efficient extrusion process capable of breaking down the agglomerates (if present) [8,17,18]. Literature study reveals that such homogeneous distribution of reinforcements can be achieved in the case of extruded materials with large deformation strain (Extrusion ratio–22.5:1), regardless of the size difference between the matrix and reinforcements [17,18].

Microstructural characterization further revealed good interfacial integrity between Mg-matrix and nano-BN reinforcement (Figure 2b) assessed in terms of interfacial debonding at the particulate-matrix interface. During processing, the oxidation of BN particulates cannot be completely ruled out, as the billet temperature reaches the near melting temperature of Mg and the formation enthalpy of B_2_O_3_ (−1262 kJ/mol) is more negative than MgO (−600 kJ/mol) [19]. The B_2_O_3_ phase (if present) will exist in the liquid state because of its low melting point (~500 °C). Further, the reaction of B_2_O_3_ with molten Mg could result in the formation of intermetallic phases, such as MgO and MgB_2_O_5_ [19,20]. The good interfacial integrity (absence of debonding and voids) at the particulate-matrix interface observed in the present case (Figure 2b) could attribute to the possible presence of oxide layer at the particle-matrix interface [21,22]. Such a good particle-matrix interface with coherent interfacial reaction products is expected to improve the strength of composite by interfacial strengthening [22]. However, the degree of interface strengthening will be greatly affected by the inherent properties of interfacial reaction products and the distribution of second phases in the matrix.

### 2.4. Coefficient of Thermal Expansion

The thermal expansion coefficients of monolithic Mg and Mg/BN composites measured in the temperature range 50 °C to 400 °C are presented in Table 3. It indicates an improvement in dimensional stability (reduction in the coefficient of thermal expansion (CTE) values) through the addition of nanoscale BN particulates. The experimental CTE values were then compared to that of the theoretical values calculated based on the rule of mixtures Equation (1) and the turner model Equation (2).

(1) $$\text{Rule}\;\text{of}\;\text{Mixtures}:\;\alpha_{c}=\alpha_{m}V_{m}+\alpha_{r}V_{r}$$

(2) $$\text{Turner}\;\text{model}:\;\alpha_{c}=\frac{\left(\alpha_{m}V_{m}K_{m}+\alpha_{r}V_{r}K_{r}\right)}{\left(V_{m}K_{m}+V_{r}K_{r}\right)}$$

where α, V and K represent the coefficient of thermal expansion, volume fraction and bulk modulus of the phase; while the subscripts, m and r, refer to matrix and reinforcement, respectively [23]. Using the values of α_m_ ~ 28.4 × 10^−6^/C, K_m_ ~ 35.6 GPa, α_r_ ~ 1.2 × 10^−6^/C and Kr ~ 145 GPa, the coefficient of thermal expansion of the composites were computed [24]. The comparison, as shown in Table 3, indicates that the experimental values were lower than both the theoretical values, as in these models, the effects of particle size on the thermal expansion coefficient has not been considered. Xu *et al*. [25] has studied the effects of varying the particle sizes and has reported that the degree of constraints posed by the particles on the matrix varies with respect to the particles sizes and, hence, affects the coefficient of thermal expansion.

**Table 3:** Results of coefficient of thermal expansion (CTE) measurements. ROM, rule of mixtures.

S. No.	Material	Thermal expansion coefficient (CTE) (× 10^−6^/K)		
		Theoretical		Experimental
		ROM	Turner model	
1	Pure Mg	28.40	28.40	28.52
2	Mg-0.5BN	28.32	28.08	27.19
3	Mg-1.5BN	28.17	27.47	26.82
4	Mg-2.5BN	28.01	26.87	24.63

### 2.5. X-ray Diffraction Studies

**Figure 3:**
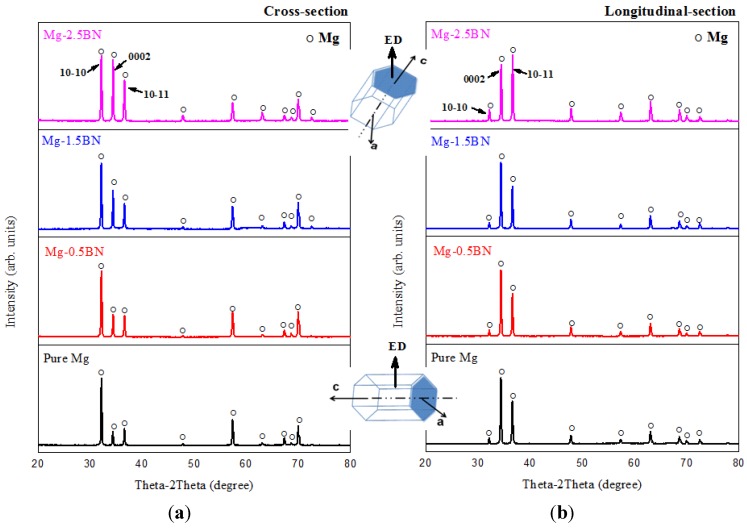
Results of X-ray characterization studies conducted along the (**a**) cross section; and (**b**) longitudinal sections on the extruded rods of developed Mg based materials.

The results of X-ray diffraction studies conducted along the cross section and longitudinal sections of developed Mg/BN nanocomposites are shown in Figure 3. The high intensity peaks corresponding to Mg were prominently seen, and the peaks corresponding to the BN reinforcement particulates and other related peaks were not prominent. This could be due to their relatively low volume fraction (<2.5 vol.%) in the Mg matrix, which would remain undetected by the technique of XRD [12]. However, the presence of these reinforcements can be confirmed from the microstructural investigation, in Figure 2.

The results of X-ray analysis were used to interpret the effect of nano-BN addition on the crystallographic orientation (specifically, the basal plane orientation) of Mg-matrix, as it is known, that the reinforcing phases could contribute to the change in basal orientation (texture) of the Mg-crystal [26]. The peaks observed at 2θ = 32°, 34° and 36° diffraction pattern of developed as-extruded Mg materials correspond to the (1 0−1 0) prism, (0 0 0 2) basal and (1 0−1 1) pyramidal planes of HCP Mg-crystal [27]. Along the cross-section, the prismatic plane intensity (2θ = 32°) is maximum in all the cases, which indicates that any of the prismatic planes is aligned in a direction, which is perpendicular to the extrusion direction. Also, the basal plane intensity (2θ = 34°) is found increasing with respect to the nano-BN addition, and in the case of Mg-2.5BN, the basal plane intensity is equally dominant, similar to the prismatic plane. Similarly, along the longitudinal section, the intensity of basal plane is found to be maximum in the case of pure Mg and the composites with a lower BN volume fraction (*i.e.*, < 2.5 wt.%). This shows that most of the basal planes are aligned parallel to the extrusion direction, indicating a strong basal texture, which is commonly observed in the wrought Mg materials [27]. However, in case of Mg-2.5BN, a change in the intensity of the peaks is clearly observed along the longitudinal section in which the intensity of the pyramidal plane (2θ = 36°) is found to be maximum. This indicates that the basal planes remain tilted and are not parallel to the extrusion direction, suggesting randomization of the extrusion texture [27]. Similar texture randomization is reported by Garces *et al.* [28] and Wang *et al.* [29], wherein it was shown that the presence of micron-sized ceramic particulates contribute to texture weakening, as seen by the reduction in the intensity of basal texture in the X-ray pole figure. Similarly, the presence of nano-BN particle reinforcements appeared to have the ability to reduce the basal intensity in the current study.

### 2.6. Mechanical Behavior

The microhardness (H_v_) values of pure Mg and its composites reinforced with nano-BN particulates are listed in Table 4. It indicates that the nanoscale addition of BN to Mg resulted in the improvement in microhardness values. The increase in microhardness values could be primarily attributed to the relatively uniform distribution of nanoscale BN particulates and its higher constraint to the localized matrix deformation during indentation [2,3]. The microhardness values of Mg/BN composites were found to be proportional to the reinforcement volume fraction and observed to be consistent with dispersion strengthened metallic materials reported in the literature [8].

The results of a room temperature tensile test conducted on the developed Mg/BN composites are listed in Table 5. From the results, significant improvement in ultimate tensile strength was observed when compared to Mg. However, considering the standard deviation, the 0.2% yield strength values were found to be similar between the monolithic Mg and its composites. In similar studies wherein the nanoscale reinforcement addition resulted in superior strength properties, the yield strength improvement is attributed to the Hall-Petch grain boundary strengthening effect offered by the increased grain boundary area by grain refinement and the presence of uniformly distributed dispersed phases, which impedes the dislocations movement [3,8,12]. While the uniform distribution of nanoscale BN reinforcements in the current study is expected to significantly improve the yield strength by effectively obstructing the dislocation movement, the minimal grain refinement would contribute less towards the grain boundary strengthening.

**Table 4:** Results of microhardness measurements.

S. NO.	Material	Microhardness H_v_
1	Pure Mg	48 ± 1
2	Mg-0.5BN	51 ± 3
3	Mg-1.5BN	55 ± 3
4	Mg-2.5BN	57 ± 2

**Table 5:** Results of room temperature tensile test.

S. No.	Material	0.2 YS [MPa]	UTS [MPa]	Fracture Strain [%]
1	Pure Mg	136 ± 8	170 ± 7	6.1 ± 1.2
2	Mg-0.5BN	127 ± 6	192 ± 8	7.8 ± 0.9
3	Mg-1.5BN	142 ± 4	200 ± 5	8.6 ± 0.5
4	Mg-2.5BN	145 ± 3	217 ± 5	7.2 ± 0.8

Further, the literature study reveals the effect of crystallographic texture modification on the yield strength properties of magnesium materials [4,7,28,29,30,31,32]. In the present study, the basal texture of composite samples was observed to be weak when compared to pure Mg, *i.e.*, the minimal basal planes intensity seen on XRD results (Figure 3) along the longitudinal sections of composite samples compared to pure Mg. In composites/nanocomposites, the addition of reinforcements usually improves the yield strength [8]. In the present case, an increase in yield strength is expected because of BN addition. However, due to the observed change in Mg-crystal orientation, the increase in yield strength is not significant. This is due to the fact that the texture weakening usually would result in the activation of basal slip at relatively lower stress levels. Such competing factors result in the yield strength values of composites being similar to pure Mg (Table 5) [31,32]. However, the presence of nano-BN particles in Mg-matrix was useful in improving the ultimate tensile strength in composites by the dispersion strengthening effect attributing to the modulus mismatch and the thermal residual stress due to the mismatch in thermal expansion coefficients between the matrix and reinforcing phases [2,3,8].

In general, extruded magnesium materials exhibit poor tensile ductility, which is attributed to the strong basal fiber texture of Mg, wherein the basal planes are aligned preferentially parallel to the extrusion direction [27]. In similar studies, the enhancement in tensile ductility was achieved by the combination of (i) grain refinement; (ii) possible activation of non-basal slip systems by the uniform distribution of nanoscale reinforcements; and (iii) texture modification [2,3,8,11,12]. In the current study, the uniform distribution of nano-BN particles in magnesium matrix as evident from the microstructural characterization (Figure 2b) would result in the activation of non-basal and cross-slip and, thereby, increase the failure strain in the developed Mg-composites [8,12,33,34,35]. Another important factor, which can be considered for the ductility improvement, is the change in crystallographic texture observed from the XRD pattern (Figure 3). It shows that most of the basal planes in the composites were titled at an angle, which is neither parallel nor perpendicular to the extrusion direction. The basal slip can be easily activated in such a case due to the slight misalignment of the basal plane as the critical resolved shear stress is very low for the basal slip to activate [12,34,35].

**Figure 4:**
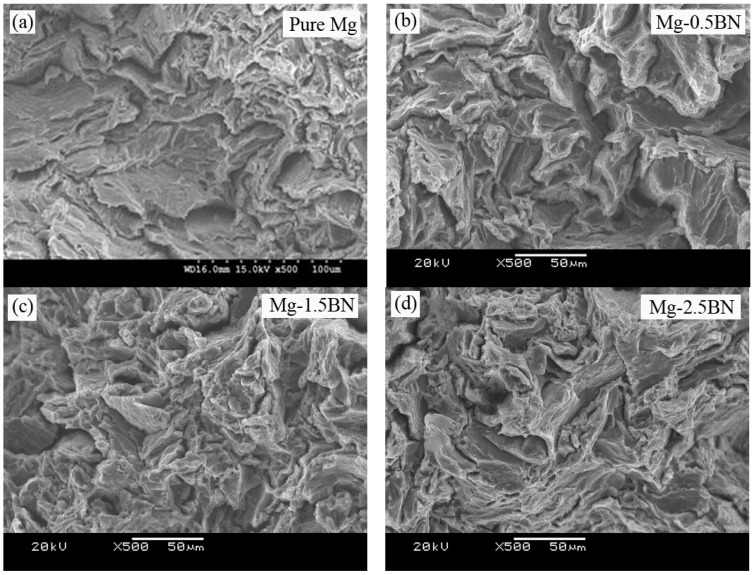

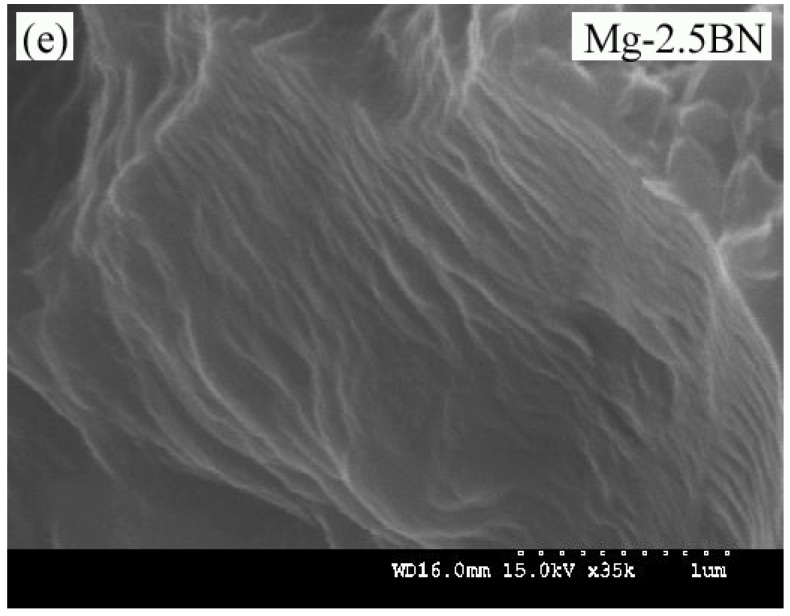
Tensile fracture surfaces of (**a**) pure Mg; (**b**) Mg-0.5BN; (**c**) Mg-1.5BN; and (**d**) Mg-2.5BN composites; (**e**) a high magnification image showing increased slip activity in Mg-2.5BN.

The fractographic evidence of the developed Mg materials under tensile loading showing the fracture mechanism is shown in Figure 4. It reveals similar fracture features, indicating the cleavage mode of fracture in pure Mg and its composites under tensile loading. From the similar fracture features observed in the fractographs, it could be understood that the fracture behavior of developed Mg/BN composites was largely controlled by the matrix deformation characteristics. However, a high magnification image (Figure 4e) shows relatively increased slip activity in Mg-2.5BN. Usually, owing to the HCP crystal structure, the plastic deformation in magnesium is limited, due to the lack of sufficient slip activity, and the cleavage steps seen in the tensile fractographs (Figure 4) are the indications of the inability of uniform deformation in Mg [5,8].

The room temperature compressive properties of developed Mg-nanocomposites are listed in Table 6. Unlike under tensile loading, where the improvement in ultimate tensile strengths due to nano-BN addition occurred with similar yield strengths (Table 5), the improvement in compressive strength properties were accompanied by a significant improvement in compressive yield strength (Table 6). In the case of extruded Mg materials with strong basal texture (with basal planes parallel to the extrusion direction), higher yield strength under tensile loading and lower yield strength under compressive is commonly observed [28,29]. This is attributed to the difference in deformation mode and the initial crystallographic orientation.

**Table 6:** Results of room temperature compression test.

S. No.	Material	0.2 CYS (Mpa)	UCS (Mpa)	Fracture Strain (%)
1	Pure Mg	70 ± 2	250 ± 7	24.5 ± 2.7
2	Mg-0.5BN	88 ± 6	290 ± 9	20.9 ± 1.8
3	Mg-1.5BN	108 ± 2	312 ± 8	19.9 ± 1.2
4	Mg-2.5BN	115 ± 4	319 ± 4	19.7 ± 1.4

**Figure 5:**
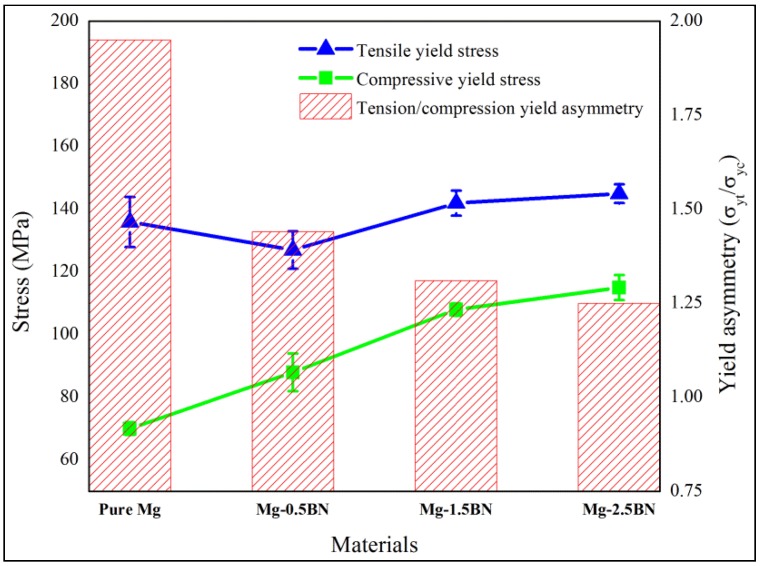
Comparison of tensile and compressive yield strengths in pure Mg and its Mg/BN composites.

**Figure 6:**
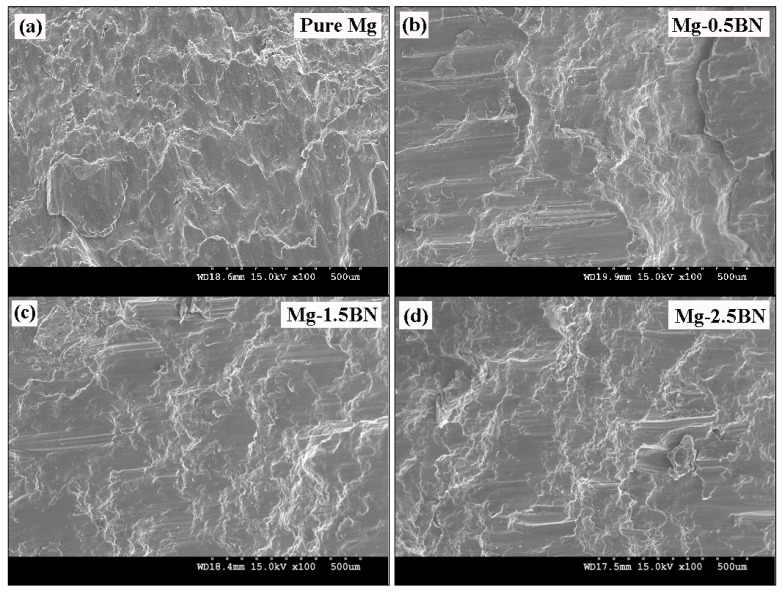
Representative compressive fracture surfaces showing shear bands in (**a**) pure Mg; (**b**) Mg-0.5BN; (**c**) Mg-1.5BN; and (**d**) Mg-2.5BN composites.

A comparison of yield strength under tension and compression is shown in Figure 5; in which the tension/compression yield asymmetric ratio is also provided. It indicates a minimal difference in yield asymmetry ($\frac{\text{Tensile}\;\text{Yield}\;\text{Strength}}{\text{Compressive}\;\text{Yield}\;\text{Strength}}$) for Mg/2.5BN composite in which the c-axis is misaligned in comparison to pure Mg, where the c-axis is parallel to the loading/extrusion axis (basal planes oriented perpendicular to the extrusion direction). The higher tensile yield strength under tensile loading often results from the difficulty in basal slip activation and the inability of twinning [7,28,29] in the case of strong fiber texture, as seen in pure Mg. Further; the formation and propagation of tensile twins were favored at relatively lower strength levels, which results in higher fracture strain, while the presence of dispersed phases in the case of composites obstructs the twin nucleation and propagation. Hence; the restricted twinning process in the case of composites delays the yield process, which contributes to the higher strength properties and the poor failure strain. The fracture surface analysis of monolithic Mg and its composites under compressive loading conditions reveals the presence of shear bands as shown in Figure 6. The presence of such shear bands indicates the twinning mode of plastic deformation, which is common in Mg alloys and composites [4,36].

## 3. Experimental Procedure

### 3.1. Materials

In this work, magnesium powder of 98.5% purity with a size range of 60–300 μm (Merck, Germany) was used as the matrix material, and nano-sized boron nitride (BN) particulates of particle size ~ 50nm (Nabond, Hong Kong, China) were used as the reinforcement. The amount of nanosized BN was varied from 0.5 to 2.5 wt.%.

### 3.2. Synthesis

Pure Mg and its nano-BN particulate reinforced composites required for the current study were synthesized using the powder metallurgy technique [15,16]. The synthesis process involved blending pure magnesium powder with different volume fractions of nanosized BN powder in a RETSCH PM-400 mechanical alloying machine (RETSCH, Hanna, Germany) at 200 rpm for 1 h. No balls or process control agent was used during the blending step, and the blending process was carried out without any protective atmosphere. The blended Mg-powder mixture was then uniaxially cold compacted at the pressure of 50 tons into billets of the size of 35 mm diameter and 40 mm length. The compacted billets were sintered using a hybrid microwave assisted two-directional sintering technique [15,16]. The billets were heated for 14 min to a temperature near the melting point of Mg in a 900 W, 2.45 GHz Sharp microwave oven using colloidal graphite as an oxidation barrier layer. After sintering, the sintered billets were coated with colloidal graphite and soaked at 400 °C for 1 h and hot extruded at 350 °C at an extrusion ratio of 20.25:1, to obtain rods of 8 mm in diameter. Samples from the extruded rods were used for further characterization.

### 3.3. Materials Characterization

#### 3.3.1. Density Measurements

The density of extruded Mg and Mg nanocomposites in the polished condition was measured using Archimedes’ principle [16]. Three samples were randomly selected from extruded rods and were weighed in air and then immersed in distilled water. An A&D ER-182A electronic balance with an accuracy of 0.0001 g was used for recording the weights. Theoretical densities of the samples were calculated using the rule-of-mixture principle. 

#### 3.3.2. Coefficient of Thermal Expansion

An INSEIS TMA PT 1000LT thermo-mechanical analysis instrument was used to determine the thermal expansion coefficients (CTE) of the as-extruded monolithic Mg and composite samples. A heating rate of 5 °C/min was maintained. An argon gas flow rate was maintained at 100 cm/min. Displacement of the test samples (each 5 mm long) as a function of temperature (50–400 °C) was measured using an alumina probe and was subsequently used to determine the CTE. The experimentally obtained values were then compared to that of the theoretical values calculated using the rule of mixtures and the turner model.

#### 3.3.3. Microstructure

Microstructural characterization studies were conducted to determine the average matrix grain size, its morphology and distribution, the presence and distribution of reinforcement and the interface between the matrix and reinforcing phase. Microstructural analyses were carried out on short sections of the extruded rod. A small section (8 to 10 mm in length) was cut from each extruded rod of different composition. Cutting was done at low speed using a diamond blade wheel cutter to produce samples with fairly flat ends. The ends of the sample were then ground using 600 and 1200 grit size sand paper to remove the large surface scratches and also to produce a flat surface. Once all visible surface scratches and cracks were removed, the sample was polished using a polishing disc with 5 micron alumina slurry, followed by 1 micron alumina slurry and lastly by 0.3 micron alumina slurry. The surface of the polished samples was etched with acetic picral (10 mL acetic acid, 4.2 g picric acid, 70 mL ethanol, 10 mL H_2_O) to make the grain boundaries visible. Acetic picral was applied onto the surface of the sample by dabbing the surface for a duration of 5 to 10 seconds, before the surface was rinsed under running water. These samples were then observed in optical microscopy and scanning electron microscopy to see the grain size, morphology and for secondary phases. The microstructures were studied using optical microscope (Olympus, Tokyo, Japan) and field emission scanning electron microscope (FESEM-S4300, Hitachi Ltd., Tokyo, Japan) coupled with energy dispersion analysis (EDS). In quantitative metallography, the characterization of the primary grain structure involves the measurement of the grain size and grain aspect ratio. Using selected optical/SEM microstructures from various compositions, the grain characteristics were determined using the Scion image analysis software. From the micrographs, using the software, the area of each grain and the values of major and minor axes of each grain can be obtained. From the values of the grain area, the average grain diameter is calculated; while from the ratio of major and minor axes, the average aspect ratio of the grains are obtained. A total of 120–150 grains were selected to calculate the grain characteristics.

#### 3.3.4. X-ray Diffraction Studies

X-ray diffraction analysis was carried out on the polished extruded Mg and Mg/BN composite samples using an automated SHIMADZU LAB-X XRD-6000 diffractometer (SHIMADZU, Kyoto, Japan). The samples were exposed to CuKα radiation (λ = 1.54056) at a scanning speed of 2 °/min. The Bragg angle and the values of the interplanar spacing (d) obtained were subsequently matched with the standard values for Mg, BN and other related phases. Further, the basal plane orientation of the developed Mg composites was analyzed based on the XRD peaks obtained from experiments carried out in directions both parallel and perpendicular to the extrusion axis.

#### 3.3.5. Mechanical Behavior

Mechanical behavior of monolithic and composite samples was assessed in terms of microhardness, tensile and compressive properties. Microhardness measurements were performed on the polished samples using a MATSUZAWA MXT 50 automatic digital microhardness tester (MATSUZAWA, Kyoto, Japan). The microhardness test was performed using a Vickers indenter under a test load of 25 gf and a dwell time of 15 s in accordance with the ASTM standard E384-99 [17].

The tensile and compressive properties of the as-extruded pure magnesium and its composite counterparts were determined in accordance with the procedures outlined in ASTM standard E8M-01 and ASTM E9-89a using an MTS 810 automated servo hydraulic mechanical testing machine [12]. The crosshead speed was set at 0.254 mm/min and 0.04 mm/min for the tension and compression test, respectively. For each composition, a minimum of 6 tests were conducted to obtain repeatable values. The fractured samples under tensile and compressive loading of Mg-materials were analyzed using a Hitachi S-4300 FESEM to identify the fracture mechanisms.

## 4. Conclusions

Mg composites reinforced with nanoscale BN particulates were successfully synthesized using microwave-assisted sintering technique, and the effects of nano-BN particulates addition on the microstructural and mechanical properties of Mg were studied. Based on the structure-property correlation, the following conclusions are drawn.

1. The addition of nanoscale BN reinforcements marginally reduced the average grain size and CTE and increased the hardness when compared to monolithic Mg.
2. XRD studies conducted on developed Mg/BN composites showed basal texture weakening with an increase in nano-BN addition.
3. Under tensile loads, the developed Mg/BN nanocomposites exhibit similar yield strength and enhanced ultimate tensile strength and ductility attributed to the strengthening effect and non-basal slip activation.
4. Under compressive loads, the addition of nano-BN particulates significantly enhanced the strength and reduced the ductility owing to the difficulty in twinning and slip dominated flow.
5. The reduction of tension-compression yield asymmetry ratio was attributed to the weakening of strong basal texture in pure Mg.
